# Sensor Technologies to Manage the Physiological Traits of Chronic Pain: A Review

**DOI:** 10.3390/s20020365

**Published:** 2020-01-08

**Authors:** David Naranjo-Hernández, Javier Reina-Tosina, Laura M. Roa

**Affiliations:** Biomedical Engineering Group, University of Seville, 41092 Seville, Spain; jreina@us.es (J.R.-T.); lroa@us.es (L.M.R.)

**Keywords:** objective pain assessment, chronic pain, body sensor networks, heart rate variability, accelerometry, skin conductance, electromyogram, image processing

## Abstract

Non-oncologic chronic pain is a common high-morbidity impairment worldwide and acknowledged as a condition with significant incidence on quality of life. Pain intensity is largely perceived as a subjective experience, what makes challenging its objective measurement. However, the physiological traces of pain make possible its correlation with vital signs, such as heart rate variability, skin conductance, electromyogram, etc., or health performance metrics derived from daily activity monitoring or facial expressions, which can be acquired with diverse sensor technologies and multisensory approaches. As the assessment and management of pain are essential issues for a wide range of clinical disorders and treatments, this paper reviews different sensor-based approaches applied to the objective evaluation of non-oncological chronic pain. The space of available technologies and resources aimed at pain assessment represent a diversified set of alternatives that can be exploited to address the multidimensional nature of pain.

## 1. Introduction

Pain is an undesired feeling that can produce a wide range of responses: from mild, localized ache to distress [1,2,3]. Despite its displeasure, it is one of the vital body alarm systems, as it allows the subject to recognize stimuli of potentially harmful intensities [4]. Pain may be located on a specific area, such as an injury, but it can also be more diffuse, as it occurs with many diseases [3]. Pain is driven by specific nerve fibers that communicate the impulses to the brain, where conscious appreciation takes place, influenced by multiple external factors [3,5].

In contrast to short-lasting acute pain, chronic pain persists even after the cessation of the initial cause and often loses its protective value [1,4,6]. By definition, chronic pain lasts over 3–6 months [7] and is not solved with treatment [8]. Its origin may be nociceptive or neuropathic [7,9], and it can be triggered by various causes (chronic musculoskeletal pain [10,11], chronic low back pain [12], fibromyalgia [13], tension-type headache [14], osteoarthritis [15], whiplash [16], heart and respiratory systems [7,17], endometriosis [18], etc.). Once developed, it may become resistant to standard treatments, greatly affecting patient quality of life [4].

Chronic pain is an important health challenge, as it represents 80% of physician visits [8]. 10% to 55% people in the world suffer chronic pain [7] (19% adults in Europe and 30% in the United States) [11]. It has been associated with great morbidity [8] and has also been proved to cause depression, neuroticism, sleep disorders, and anxiety [7]. Among the elderly population, it is a common impairment with a significant impact on physical and psychological state, quality of life, and even a barrier to social inclusiveness [19].

Pain assessment and management are essential issues to provide interventions to a wide range of disorders [1,20]. However, as pain is a subjective experience, its measurement is difficult [21]. Sensitivity to pain is manifold in the different individuals [22], in part due to its association with motivational and emotional factors (depression, anger, anxiety, resentment), behavioural components, the influence of life-style (marital or workplace distress, etc.), personal skills, capacity to face pain, health beliefs, etc. [3]. Pain is a very personal sensation that is difficult to analyze, understand and explain, moreover if there is a lack of proper communication with the patient [23].

The commonly accepted method to measure pain in adults is based on self-reports and subjective reports from patients, caregivers or medical staff [20,21]. These reports are frequently complemented with physiological measurements, behavioural observations, interviews, medical records, and psychometric measures, among others [3].

**Figure 1:**
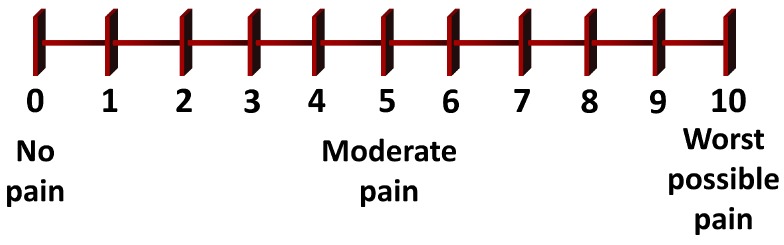
Visual analog scale (VAS) pain scale.

The visual analog scale (VAS) and McGill pain questionnaire (MPQ) are the most popular self-evaluation instruments to measure pain intensity in clinical and research environments [3,24,25]. VAS is a graphical method that requests the respondent to self-evaluate her/his pain in a 10cm-length horizontal line with diverse pain legends, with scores ranging from 0 (there is no pain) to 10 (the worst possible pain) (see Figure 1). MPQ defines a questionnaire with words grouped into 20 subclasses [26]. The interviewer asks the respondent to choose the word in each class that better matches her/his pain. If no word defines the pain in a class, he/she will not make a choice. Each word has a value, and the overall summation provides the pain rating index (PRI). Present pain intensity (PPI) is obtained by combining the numbers of the chosen words.

While these tools are convenient and useful, subjective reports present several limitations [20], as they include inconsistent metrics, are affected by reactivity to suggestion, efforts in the control of impression or deception, and are prone to differences in the conceptualization of pain represented by the medical staff and patients. Moreover, these methods cannot be applied to children or patients with neurologic disorders, dementia or transient states of consciousness [20].

An inaccurate and inconsistent assessment of pain can lead to an inappropriate and insufficient therapy [27]. Consequently, instead of relief, the patient can experience a physical worsening, physiopathologic effects (increase of blood pressure, heart rate, etc. [19]), and exacerbate her/his psychological distress [27].

A better assessment of pain would allow the personalization of diagnostic and treatment interventions in chronic pain disorders [28], and it is essential to identify novel therapeutic approaches [4]. The objective inspection of pain has received a great attention during the last years, mainly due to the benefits that it can provide to healthcare (e.g., the election of the most appropriate therapy to improve the quality of life [27], patient follow-up in intensive care units [29], reduce an over-stimulation in rehabilitation, optimize the administration of anesthesia [30] and the dosage of analgesics [23]), and in particular to cases where patients cannot describe their pain in the clinical context and daily life (drowsiness, dementia, etc.) [23]. For these reasons, the accurate measurement of pain is still a challenge for researchers, healthcare professionals, the legal community and patients with chronic pain [28].

The objective assessment of chronic pain is a difficult problem that can be of great interest in multiple clinical areas to procure a better patient intervention and an improvement in the quality of life of chronic patients of different etiology. It has been conducted in the bibliography from different points of view, ranging from video signal processing [29,31,32] or the analysis of bio-physiological signals [33,34,35,36], to systems combining heterogeneous information sources [3,11,27,37,38,39,40]. However, to date a universal method has been developed neither for the objective evaluation of pain [22], nor for the measurement of its seriousness, due to the subjective nature of pain perception by the sufferer, although it is a critical demand for multiple disorders. In the absence of objective measurement reference standards, the evaluation is carried out through subjective self-reports from patients, caregivers or medical staff. It should be noted that despite the lack of an objective reference for the evaluation of chronic pain, many studies have tried to induce pain levels in a controlled manner (by heat [23,41,42,43,44,45,46,47,48,49,50,51,52,53], cold [45,48,52], pressure [44] or electrical stimulation [54]) as a first approximation to a method that allows the evaluation of chronic pain [1]. In contrast to the studies based on research with phasic pain stimuli (short duration), the number of studies based on tonic pain stimuli is growing as an approach to chronic pain [23,41,42,43,44,45,46,47,48,49,50,51,52,53,54,55,55]. This validation approach represents an objective way for assessing chronic pain, leaving aside all the subjectivity that may be related to the perception of pain by the chronic patient [23,41,42,43,44,45,46,47,48,49,50,51,52,53,54,55,55]. In addition, it becomes the most appropriate option in the case of patients with absence of communication skills, whether verbal, written or tactile (infants, unconscious patients, critically sick, patients with intellectual impairment, advanced dementia, etc. [45]). Therefore, and given the importance and benefits of the objective evaluation of chronic pain for the scientific community in general, and patients in particular, a great research effort must be carried out from multiple technical and clinical points of view, given the interdisciplinary nature of pain assessment. In this sense, the present work provides a comprehensive overview of sensor technologies and physiological aspects related to the underlying technologies for chronic pain assessment, whether of musculoskeletal, gastrointestinal, fibromyalgia, arthritis, migraine, or other types of chronic pain of non-oncological origin.

Regarding the technological alternatives for pain assessment, it is worth mentioning that portable body sensor technologies have experienced a rapid growth in recent years [56,57,58,59,60,61]. These technologies allow remote monitoring of biomedical parameters (body temperature, heart and respiratory rate, physical activity, etc.) in a non-obstructive way thanks to the wireless communications of the body sensor networks (BSN) [62,63,64,65,66,67]. As the next sections report, some of these signs can be used for the objective evaluation of pain. On the other hand, numerous investigations have been carried out to incorporate BSNs in healthcare not only for the remote monitoring of patients, but also due to the advantages offered by biomedical data processing methods and techniques and information fusion from different sensors applied to the early detection of diseases and health adverse events [68,69,70,71,72], including pain management. In addition, increasing the awareness of citizens about their own health state as a result of the use of sensors allows them to take a more proactive attitude, favoring self-care and promoting healthier habits and lifestyles. BSNs are thus contributing towards a paradigm shift from a curative and reactive medicine, to a more preventive, proactive and personalized medicine [62,63,64,73,74,75].

This paper reviews the different methods described for the objective evaluation of pain. In particular, it is focused on non-oncologic chronic pain. Section 2 describes the relation and use of heart rate variability for the analysis of chronic pain. Section 3 reveals the interest of accelerometry and its application to the evaluation of physical activity in the study of chronic pain. The utility of skin conductance for the evaluation of pain is analyzed in Section 4. Section 5 reports different examples of the use of biopotentials to analyze chronic pain. Different computer vision techniques have also been employed for pain recognition and Section 6 reviews some significant examples from the literature. Section 7 compiles other sensor technologies that have been applied to the assessment of chronic pain and analyzes the benefits of data fusion. Section 8 desribes processing algorithms and computational models for applied to pain assessment. Section 9 reports some examples of digital resources (databases and mobile apps) that have been developed in the context of chronic pain detection. Finally, Section 10 draws the conclusions and future research lines.

## 2. Heart Rate Variability and Chronic Pain

In the ancient Greece, Aristotle suggested that pain was perceived by the soul, which was located in the heart [76]. Today, it is indisputable that the nociceptive information is processed by the brain, however, extensive interactions between the neural structures involved in pain feeling and heart autonomous control can be observed [77]. As heart rate is affected by emotional activity, this indicator can be used to distinguish between positive and negative emotions [78,79].

The autonomic nervous system (ANS) plays a key role in the regulation of heart rate [79,80]. The parasympathetic nervous system makes heart rate and in turn blood pressure to decrease, whereas the sympathetic nervous system favors the antagonistic action, increasing heart rate and blood pressure.

The sympathetic system prepares us to face situations requiring energy expenditure: an emergency threatening the body internal equilibrium, a sudden and intense exercise, an aggression or temperature change, stress situation, etc. The parasympathetic system stimulates activities that take place in normal conditions to ensure long-term wellness (e.g., digestion) and ease energy storage or saving. Both systems are antagonistic and may not be activated simultaneously, such that the action of any of them “restrains” the other. The interaction between these systems is known as the sympathetic-vagal balance of the ANS [80].

**Figure 2:**
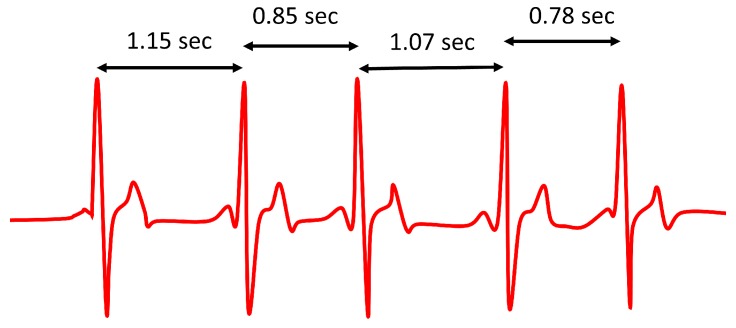
Variations of R–R intervals in an electrocardiogram (ECG) signal.

A sign of ANS balance and its conditions is heart rate variability (HRV) [81,82,83,84], which refers to short- and long-term variations of heart rate due to several causes. HRV records the variation of beat-to-beat time intervals, also known as R–R intervals, as heart beats are usually associated with the R wave of the electrocardiagram (ECG) [82,85]. An average heart rate of 60 beats per minute does not mean that the interval between successive heart beats is exactly 1.0 s, since it can experience variations in the range between 0.5 s and 2.0 s, with HRV indicating the fluctuations of heart rate around its average value (see Figure 2).

The ECG signal is an expression of the contractile activity of heart, which can be registered on the breast surface or limbs. Although recording ECG in the limbs presents less drawbacks, it is more sensitive to artifacts [43]. From ECG signals, HRV is measured using long- and short-term recordings. The former use to last 24 hours and are acquired with a Holter, while the latter take 2 to 5 min and are based on a dynamic ECG [86]. Although HRV is commonly established through ECG, it can also be obtained with photopletismography [87].

**Figure 3:**
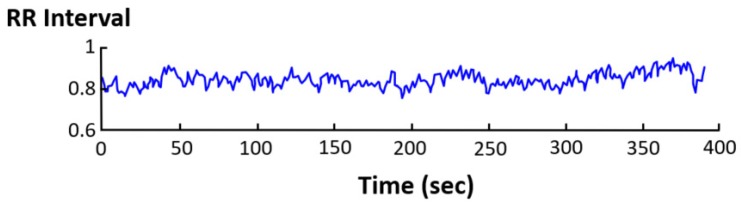
Example of a signal obtained from ECG R–R intervals.

Time-domain and frequency-domain methods can be distinguished to measure HRV. The former are based on the statistical analysis of the time distance between R waves of the ECG signal (R–R intervals, see Figure 2 and Figure 3). However, their capabilities are limited in terms of specificity and sensibility, and they require long recording periods [86].

**Figure 4:**
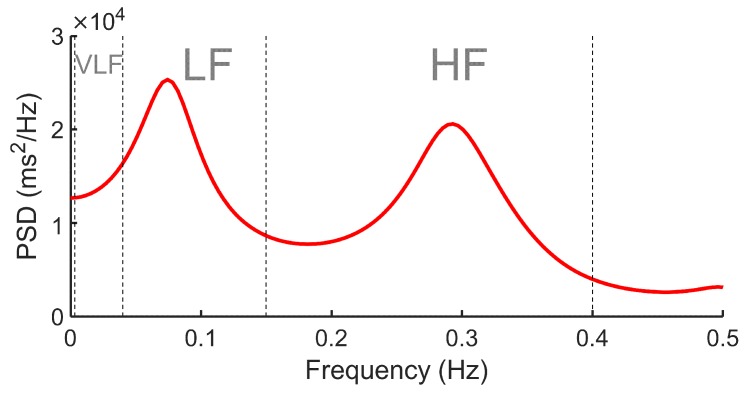
Spectrum of a HRV signal.

As aforementioned, heart rate is not fixed and some ANS-related processes induce variations that are oscillatory or repeated periodically. Frequency-domain methods or HRV spectral estimation identify the spectral components (oscillatory processes) implicit in HRV [82]. To that aim, an estimation of power spectral density (PSD) of the HRV signal is usually performed. As Figure 4 shows, the range between 0.0033 and 0.04 Hz identifies very-low-frequency (VLF) spectral components [88]; the 0.04–0.15 Hz range identifies low-frequency (LF) components; and 0.15-0.4 Hz are associated with the high-frequency (HF) range [89]. These components provide clinical information about the variation of heart sinus rhythm. The LF components are influenced both by the sympathetic and parasympathetic system, although they can also be associated with baroreflex sensitivity [90]. The HF components are related to the parasympathetic system, hence the LF/HF ratio provides information about the sympathetic-vagal balance [90].

HRV may be affected by situations requiring a mental effort or stress [19]. A low HRV can be associated with a relaxing state, while the increase of HRV is an indication of potential stress, pain or mental frustration [43]. The vagus nerve influences pain modulation [91] in such a way that chronic pain is associated with an alteration of the descendent inhibitory pathway. As HRV is an approximate sign of of vagal activity, this variable can be related to a dysfunction of the descendent inhibitory pathway. Results reported in [91] support this effect. There are many studies relating chronic pain of different etiology (headache, neck, shoulder, lumbar, hip, etc.) with a dysfunction of the ANS, and specifically with a low HRV mediated by a reduction of the parasympathetic cardiac activity [77,92,93,94,95,96,97] and a sympathetic activation [91]. This relation has also been evidenced in trials with animals [98].

As the frequency components of HRV allow a detailed analysis of the sympathetic–vagal balance, its study is useful for the evaluation of chronic pain. In [99], a method was developed to assess the intensity of pain through a spectral analysis of HRV fluctuations [100]. An analysis of 51 trials evaluating the influence of chronic pain in the HRV spectrum [95] showed a consistent (moderate to high) effect of reduction of the HF component of HRV in patients with chronic pain, what implies a reduction of parasympathetic activity. This effect is even more obvious in trials addressing fibromyalgia. As aforementioned, persons with chronic pain have a reduced HRV compared with no pain, with differences specially relevant in the HF component [77,95].

Next, results reported in different trials about the assessment of pain through HRV are grouped depending on the etiology of condition, and its application to diverse chronic pain treatments.

### 2.1. Pain of Musculoskeletal Origin and HRV

The analysis reported in [22] using HRV reveals a reduction of the parasympathetic activity and an increase of the sympathetic activity in patients with chronic pain of musculoskeletal origin.

According to [101], a reduction of cervical chronic pain is associated with a reduction of the LF component of HRV, an increase of the HF component and a reduction of the LF/HF ratio.

In [102], a trial with patients suffering from shoulder and neck pain reported a significant reduction of the LF/HF ratio after the execution of isometric exercise in the control group, while no significant changes were observed in patients with no pain.

In [103], a trial with patients suffering form sciatic pain referred to backbone surgery showed a higher LF/HF ratio in the control group. Moreover, in these patients a higher HF component was correlated with a higher duration of pain episodes.

### 2.2. Fibromyalgia and HRV

Fibromyalgia is a chronic pain syndrome characterized by a generalized musculoskeletal pain, painful sensing to pressure in specific points, fatigue, unrelaxed sleep and depressed mood [94]. HRV has been correlated with pain in patients with fibromyalgia and it is sensitive to changes of condition, with potential of becoming a useful biomarker for the diagnosis of this disease [94].

### 2.3. Gastrointestinal Pain and HRV

HRV is a commonly used index in gastroenterology, as it reflects the unbalance between the primary excitatory and inhibitory body systems [104]. ANS is the main communications pathway between brain and intestine, hence it is of particular interest for gastrointestinal disorders, as it is the case of irritable intestine syndrome, which is characterized by a functional abdominal pain [105]. Defining the gravity of the pain according to its duration and intensity, a lower HRV has been related with a higher gravity of abdominal pain in these patients [28,106,107].

Irritable intestine syndrome was also analyzed in [105]. Results showed a significantly lower HRV in women with persistent pain in comparison with women with controlled pain or no pain, and also with respect to men, independently of their pain state. A spectral analysis of HRV showed a reduction of the HF components, i.e., a reduced parasympathetic “restrain” of the sympathetic activity, either in rest and during stress episodes. This phenomenon can be reflecting a peripheral mechanism, such as an intestinal dysfunction, or a mechanism of the central nervous system, such as pain amplification or deficient emotional self-regulation.

According to [108], a higher duration of the disease (chronicity) is related to a reduction of the LF component of HRV, and a higher seriousness of abdominal pain is related with a decrease of the HF component.

### 2.4. Burning Mouth Syndrome and HRV

The analysis of HRV reported in [109] highlighted the autonomic instability present in the burning mouth syndrome, a chronic pain characterized by a burning sensation in the tongue or mouth. The application of (near infrared) radiation in the stellate ganglion fixes the autonomic dysfunction, being detectable by HRV, what can be useful to follow the evolution and evaluate the therapeutic effectiveness of this approach.

### 2.5. Pain Catastrophizing and HRV

Pain catastrophizing is “characterized by the trend to magnify the threatening value of pain stimuli and vulnerable feeling in the context of pain, and a relative incapacity to inhibit thoughts related to pain before, during or after a painful episode” [110]. According to [111], pain catastrophizing is inversely related to the HF components of HRV, which could be used as an index to assess the capacity of emotion regulation and perception of pain threat. The reduction of the HF components of HRV can be the psychophysiological mechanism underlying a higher pain catastrophizing.

Similar results were reported in [8], where pain anxiety and feelings were rated as significant predictors of HRV in a group of patients with benign pain, including patients with fibromyalgia, exposing its relation with chronic pain and autonomic function.

### 2.6. Pain Therapies and HRV

**Figure 5:**
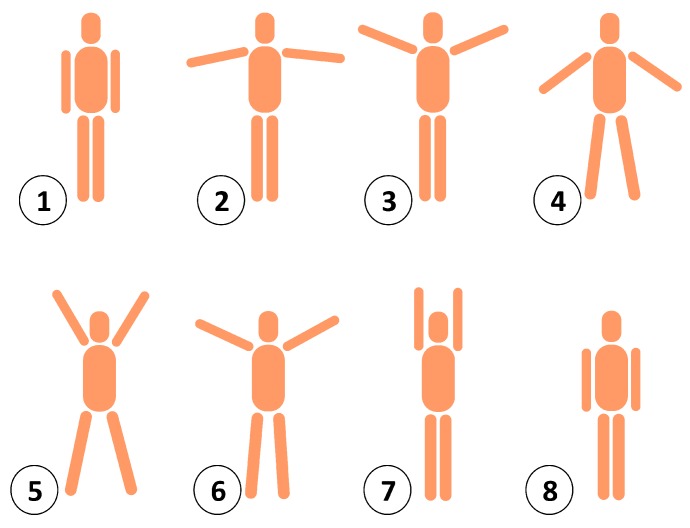
Sequence of movements in anthroposophic therapy.

Results obtained in [112] show that an anthroposophic therapy can be beneficial to several conditions, such as chronic pain, hypertension and mood disorders, with an impact on HRV (see Figure 5).

Biofeedback therapy is a trans-disciplinary intervention method for chronic pain [113]. These techniques provide patients with visual or auditive feedback of information in real time related to a biological signal reactive to pain and unknown to the patient [114]. Frequently used biomedical signals are electromyogram recordings from a target muscle, temperature, HRV, respiratory frequency or skin galvanic response. The objective is that the patient can perform an intentional control of the biomedical signal, in order to interrupt the reactivity to pain. Results obtained in [114] show that the use of HRV in biofeedback therapies provide benefits in different measurements of reactivity to pain.

The percutaneous stimulation of auricular vagal nerve has been demonstrated to modulate the body sympathovagal (autonomous) balance, becoming an effective treatment for depression, epilepsy and acute and chronic pain [115]. A trial with primary cervical dystonia patients [116] revealed that the stimulation of the vagal nerve led to a reduction of muscle pain, dystonia symptoms and a subjective recovery of motility, sleep and mood, situations that were correlated with an increase of HRV median from 37.8 to 67.6 ms. The spectral analysis of HRV showed a decrease of HF components, what indicates a higher activity of the parasympathetic activity.

Cervical manipulation, often used for the treatment of musculoskeletal disorders, such as neck pain, has a significant influence on HRV, increasing the parasympathetic response and decreasing the sympathetic tone [117].

In a study conducted with patients suffering from chronic lumbar pain (the most prevalent musculoskeletal disorder [118]) associated with an altered alignment of intervertebral discs [119], the practice of Yoga during three months reduced pain feeling, the LF components of HRV and respiratory rate, and increased significantly the HF components of HRV. Mindfulness-oriented recovery enhancement (MORE) integrates training in “tasting” natural rewards with mindful training and cognitive re-evaluation. It has a demonstrated effectiveness in the reduction of chronic pain, the misuse of opioids and the desire of being in contact with social support groups [120]. The application of this technique has been associated with an increase of HRV [121].

On the other hand, results reported in [122] reveal that HRV can be an useful index for patients suffering from chronic pain that misuse prescribed opioids to procure a better self-regulation and response to the dosage.

## 3. Accelerometry-Based Activity Evaluation and Chronic Pain

Another way of identifying the presence of pain is through movements and physical activity, which are essentially different in subjects with chronic pain [25]. It is commonly considered that people who feel impaired and report restrictions in daily-life due to pain are less physically active [123,124,125]. People with chronic pain of physical origin adopt specific behaviors in response to pain, or when this is expected to occur, to protect the body [11,124]. Anxiety produces a feeling of pain augmentation and/or a higher damage makes people behave in a self-protecting way, e.g., avoiding a particular movement. For example, patients with chronic lumbar pain show a coordination of movements and activity patterns different from healthy subjects [126]. These use to move hip and knees asynchronously when performing a lifting exercise, while patients with lumbar chronic pain use to move hip and knees simultaneously [127]. In [128], the variation of shoulder acceleration during lifting movements was evaluated, and the rate was lower for subjects with pain. Patients who have neck pain have a tendency to move the trunk with less intensity, performing the activities more slowly [129].

Pain responsiveness conducts include protection, hesitation, stiffness and fastening. Four subgroups representing the resistance-avoidance pain regulation response model patterns were analyzed in [130]: resistance to distress, resistance to stress, avoidance of apprehension (to pain) and adaptive responses. Results showed a higher pain, impairment and fatigue in patients belonging to the apprehension avoidance group compared to patients with adaptive responses and, as expected, a lower physical activity. However, despite all these conditions, the measurement of physical activity is a challenge for the treatment, rehabilitation and promotion of healthy habits in patients suffering from non-oncologic chronic pain [124,131,132].

The common methods applied to the evaluation of physical activity in persons with chronic pain are self-evaluation questionnaires, such as the international physical activity questionnaire or Baecke physical activity questionnaire (BPAQ). However, these methods have shown a lack of agreement regarding the estimates of physical activity obtained from sensor devices [133,134,135,136]. Moreover, patients with high levels of depression use to underscore their activity level [135].

This scenario justifies current efforts for the development of objective physical activity evaluation systems for the identification of pain-related activities (breast pain, headache, etc.) [137,138], for the evaluation of pain intensity [139,140,141,142,143,144,145,146,147], for the detection of postures, movements or activities that can produce pain [148,149,150,151,152,153], or for the improvement of quality of life and a reduction of pain intensity [154,155,156,157,158].

**Figure 6:**
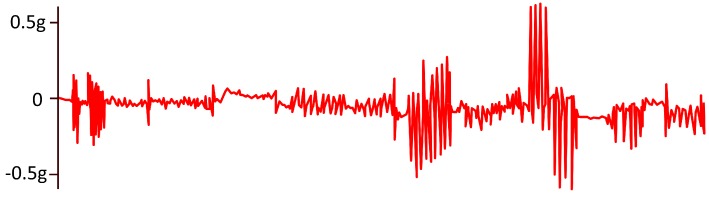
Example of uniaxial acceleration acquired with a physical activity monitoring device.

Accelerometry is the common method for the objective evaluation of physical activity in observational studies [159] (see Figure 6). There are several accelerometer-based wearable systems for the continuous monitoring of daily activity, as they allow recording variations in the orientation and an easy detection of movements [160]. Some authors have proposed using the count of accumulated steps as an estimate for physical activity [140]. Other studies refer to the total daily activity minutes [161] or a measurement of the intensity of activity [162]. Most traditional accelerometer devices only record a measurement proportional to the average acceleration in a time lapse [159,163], what may lead to errors in the classification of activity when compared with the reference method [164]. Other proposals have been suggested based on the frequency of postural transitions [165] and estimation of energy expenditure [166]. Nevertheless, the systems reporting more information about the behavioral patterns and habits of the monitored persons are the daily life activity classifiers [167,168].

Next, some results related to the evaluation of pain through accelerometry and the study of physical activity are detailed.

### 3.1. Physical Activity as a Predictor of Pain

The origin of musculoskeletal pain is often associated with inappropriate postures or movements [148,149,169]. Such is the case of the study reported in [150], which suggests a relation between the sitting time during the full day and specifically during work time, and the intensity of neck/ shoulder pain in workers developing manual work. In a similar group of persons, a negative correlation was established between walking time during work and lumbar pain [151].

On the contrary, in health sector workers, the amount of sitting time is inversely related with lumbar pain intensity [152]. In another working area, results reported in [153] showed that grape-pruning activity leads to the adoption of curved and forward folded trunk postures inducing a significant risk of lumbar musculoskeletal disorders.

The work presented in [169] describes a system for the prevention, diagnosis and treatment of neck pain in office workers. The referred system is formed by neck movement sensors and a smartphone that allows system management and feedback with the patient to correct inappropriate postures. The authors of the system proposed in [124] based on portable motion sensors investigated the automatic detection of self-protective behaviors.

### 3.2. Physical Activity as a Pain Index

Several trials have suggested the use of physical activity as an objective index for pain, based on the fact that the intensity of average movement, the maximum intensity of movement and postures acquired through accelerometry are different in persons with chronic pain compared to healthy persons [139]. Results published in [140] show that it is possible to discriminate patients with complex regional pain syndrome from control subjects using a small set of gait characteristics extracted from accelerometer data during a short set of physical performance tests [170]. In [24], gait using inertial sensors was analyzed in adult subjects with chronic arthralgia after Chikungunya virus infection, finding a correlation of pain with alterations in gait, grip strength and balance.

The intensity of physical activity evaluated through accelerometry has also been applied to the assessment of the intensity of chronic or recurrent sub-acute lumbar pain in adolescents [141], in patients with hip or knee osteoarthritis [142], shoulder pain in persons with type-2 diabetes [143], the detection of walking impairments after abdominal surgery [144], juvenile idiopathic arthritis [145], or chronic pain of musculo-skeletal origin in patients and workers [146,147].

In [171], an abdominal pain detection system is proposed based on the accelerometers integrated in a smartphone. In this system, the patient reproduces in a guided way the palpation movement using the smartphone. A detection algorithm recognizes pain events from the acquired accelerometer information.

Pediatric abdominal pain has also been evaluated by means of accelerometry through the assessment of the sleep and physical activity [172]. The relationship between self-scored pain, muscular activity and postural load during cleaning activities was studied in [173], and the authors found a higher muscular activity in the case of low-level pain compared to subjects reporting a higher level of pain.

Accelerometry has also been used to study the relationship of physical activity with pain in patients with knee osteoarthritis, since these patients may become more inactive due to pain and functional limitations [174]. Conversely, the results obtained in [25] indicated an increase in physical activity in patients with osteoarthritis of the facet joint after a paravertebral spinal injection.

In [175], the authors made an objective comparison of physical activity in patients with chronic lumbar pain, who were classified in several groups: avoidance, persistent, mixed executors (significant avoidance and persistent behavior), or functional executors (reduced avoidance and persistent behavior). Physical activity level did not differ among the four groups. However, an association between the pain intensity level and the activity level in the avoidance and persistent groups was found.

In addition to activity intensity, pattern activities experience detectable alterations in persons affected by pain. According to [176], the distribution of activities during the day significantly differ in patients with chronic lumbar pain. This study reported that these patients show higher activity levels during the morning and lower during the night, compared to control subjects.

Although physical activity has been usually associated with lower levels of pain (the lower the activity the lower the inhibitory capacity of pain, and the higher activity the lower pain [177]), some conditions are characterized by the opposite. In persons with knee osteoarthritis, the increase of knee load frequency (steps/day evaluated through accelerometry) and its magnitude (knee adduction angular impulse evaluated through isokinetic dynamometry) has been associated with an increase of pain [178]. The temporal relationship between pain and activity in patients with acute lumbar pain was studied in [179]. According to their results, a high correlation between activity and pain was evidenced during the first week of acute lumbar pain. In most cases, pain persisted 30 min after activity. As patient condition improved and reported less pain, the relation between activity and pain disappeared.

### 3.3. Benefits of Physical Activity to Pain

Physical activity has also been related to an improved quality-of-life and a reduction of pain intensity in patients with chronic pain. The reduction of pain through physical activity has been shown to be able to improve the ability of people with musculoskeletal pain to perform daily activities [180].

In [154], the authors analyzed physical activity performed by patients with fibromyalgia. Participants in the trial mostly had sedentary habits, characterized by 4019±1530 steps/day. Results showed a linear relation between the increase of day steps and health results.

Another study showing the benefits of performing moderate physical activity for the reduction of neck/shoulder pain in workers and manual labor was reported in [155].

Physical activity has been associated with an improved quality-of-life in persons with multiple sclerosis [156]. This work concluded that the most physically active patients reported lower levels of disability, depression, fatigue and pain.

The study developed in [157] associated a low or moderate physical activity with reductions in the verbal rating scale pain assessment for patients enrolled in a cognitive-behavioral therapy intervention program.

The results reported in [158] suggest that the development of highly intense activities and a high rate of activity fluctuations are associated with a worse sleep in patients with chronic pain. Therefore, the modulation of activity may be a key therapy to address sleep disorders in subjects with chronic pain.

Considering that the benefits of physical activity are dependent on their correct performance, the control of adherence to activity programs at home is of great interest, which can also be evaluated through accelerometry [181]. This is also the purpose of the “selfBACK” m-Health decision support system, which is introduced in [182,183] for the self-control of lumbar pain. This system controls the activity of the subjects through human activity recognition by using accelerometer sensors to evaluate their adherence to the prescribed physical activity plans.

The authors of another work [184] used an inertial sensor to evaluate the angular velocity and the maximum angle of column movements. Their results confirm the possibility of using this information for a quantitative evaluation of the rehabilitation progress of patients with low back pain.

## 4. Skin Conductance and Chronic Pain

Skin conductance is a parameter related to the electrical resistance of skin, which increases with sweat [19]. Electrodermal activity is considered a sign of internal tension of a subject, as sweat glands are innervated by the sympathetic branch of the ANS. In presence of a stress situation, pain, emotional excitation or an intense mental effort, skin conductance can experience a sudden increase in a period of 1–3 s. This signal allows the differentiation between conflict and no-conflict situation, or between rage and fear.

Conductance measurement is based on the injection of an electric current into the body through a pair of electrodes, and the measure of the electric voltage produced as a consequence of the circulation of electric current through the body [186]. According to Ohm’s law, the ratio between voltage and current provides an impedance (Z=V/I). This way, impedance (*Z*) is described as the opposition of a conductor to the electric current flow. This property is dependent on frequency and the particular characteristics of the medium through which current flows, and it is inversely proportional to conductivity (σ).

**Figure 7:**
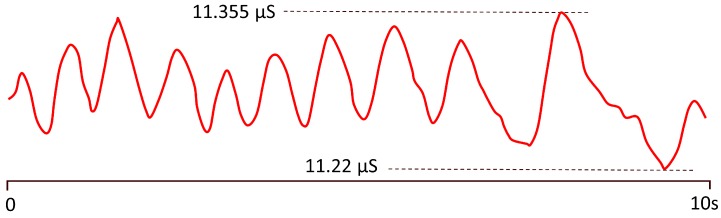
Fluctuations of skin conductance registered in [185].

Results published in [41] show a relationship between the response of skin conductance and pain induced by heat. A study focused on the evaluation of post-surgery pain evidenced a correlation between the number of fluctuations per second of skin conductance and the level of pain (0.07 for no pain, 0.16 for low pain, 0.28 for moderate pain y 0.33 for severe pain) [185,187] (see Figure 7). The problem is that this signal is also influenced by factors such as external temperature, and requires reference measurements and calibration [43].

Skin conductance has also been used for the evaluation of pain in patients with fibromyalgia [188,189,190], showing a response above the base line [191,192], and also for the evaluation of pain in patients with chronic pain in the back [193,194,195,196,197,198] or with a high risk of chronic back pain [197], for the evaluation of shoulder pain [199], chronic lumbar pain [200], patients with post-trauma stress disorders [201], higher depressive disorder [202], tensional headache [195,203], migraine [204], irritable intestine syndrome [205], or arthritis [196]. The authors of [206] quantified the response to pain in patients with Rett syndrome by measuring skin conductance.

In [207], the electrodermal reactivity of patients with chronic lumbar pain to emotional speech was analyzed. In these patients, the response of skin conductance induced by pain-related speech was higher compared to neutral speech. This sensitivity to pain-related speech was also evidenced by the study developed in [208]. Similar trials were conducted in [209] and [210], by analyzing reactivity to subjective thoughts of pain perception [209] or video images [210], finding equivalent results.

Patients with chronic pain exhibit impaired cognitive functions, affecting decision making [211]. In the group of patients studied in [212], an association was reported between the lack of an anticipatory response of skin conductance in decision making process, what suggests that these processes are more dependent on cortical resources.

On the other hand, slow and deep respiration techniques, used in diverse relaxation methods for the treatment of chronic pain [213], have reported a significant effect in the reduction of skin conductance [214].

## 5. Biopotentials for Pain Assessment

The electrical biopotentials generated by the human body are signals commonly used in medical diagnosis. The main biopotentials are electrocardiagram (ECG) [82,85], electromyogram (EMG) [11,35,43,215] and electroencephalogram (EEG) [46,50,216,217,218,219]. Biopotential sensors are usually composed of the following elements: electrodes, used for the transduction of the ionic signals inside the body to electrical signals; an analog conditioning stage for the amplification of the electrical signal, of very low intensity, at measurable levels avoiding electromagnetic interference (EMI); and an analog–digital conversion stage. Isolation barriers to protect the physical integrity of the user are necessary. Research about the biopotential instrumentation is focused on obtaining greater patient comfort, an improvement in portability and ease of use. A problem presented by this type of systems is that the electrodes produce drifts of the continuous level that can be several orders of magnitude above the amplitude of the signal, such that a high amplification gain can saturate the signal. The classic solution is based on analog electronic stages applying high-pass signal filtering, distributing the amplification in several stages, one with lower gain before filtering and a final stage of high gain for the adaptation to adequate signal levels. At present, sigma–delta analog–digital conversion technology provides very high accuracy, which reduces the need for gain of the amplification stage.

### 5.1. Electromyogram Sensors and Chronic Pain

Muscular electric activity is a sign of general psychophysiological activity, in general [35], and in particular, an increase of muscular tone reveals an increase of the sympathetic nervous system, whereas a reduction of somatomotor activity is associated with parasympathetic activation [43]. A high level of muscular tension is a sign of stress [220], which is also expected under a pain situation [35,43].

EMG is a measurement of biopotentials related to the muscle electric activity, which is related to muscular activity or the frequency of muscular tension [220]. For this reason, EMG signals have been used in pain assessment studies [11]. Low back pain is related to alterations of the muscular function of the trunk as a consequence of pathological states of muscular activation by the central nervous system. The measurement of the surface electromyogram (sEMG) has been applied to the study of these alterations [221]. The EMG measurement system proposed in [222] evaluated neck fatigue by providing the user with feedback for posture correction [222]. EMG has also been applied to the evaluation of low back pain [215]. In [223], shoulder and neck pain induced as a physiological response to stress were shown to be associated with a slight increase of heart rate and sEMG activity at the trapecius muscle. In [224], an absence of muscular relaxation response in the flexion movement of the trunk was reported for persons with chronic pain. Moreover, in these subjects a lower muscular activation was evidenced in the contraction phase. As no differences were found in the muscular activation patterns when they did not move, this study suggested the possibility of identifying chronic pain during the execution of physical activities.

Another study recorded the EMG of maxillar muscles in patients with disorders in the temporoflexion exercises, analyzing the flexion/exmandibular joint, showing a higher activity for mandibular activity during rest periods in patients with pain compared to healthy subjects [225]. In [226], EMG was used in tension ratio for the activation of the lumbar para-spinal muscle, the muscular activation for the medium lumbar para-spinal muscle, and the total flexion developed. The use of these indices provided a classification of subjects with pain with a precision of 86%.

In [118], an electrode matrix was used for the acquisition of EMG signals and a Bayesian classifier was proposed as a predictor of lumbar pain (70% success). In this case, Sorenson test was applied to induce muscle fatigue in the lumbar region [118]. The same technique was used in [227], but in this case with an artificial neural network (80% success).

EMG signals were applied in [228] to detect patients with chronic neck pain through the analysis of nociceptive withdrawal reflex in the leg. Nociceptive withdrawal reflex has demonstrated to be a useful tool for the objective evaluation of hyperexcitability of spinal chord present in chronic pain disorders. Under a pain situation, the nociceptive system experiences a change leading to hyperexcitability, i.e., to the presence of pain after innocuous stimulation, or excessive pain after low-intensity nociceptive stimulation. Patients with chronic pain syndrome, such as cervical stabbing syndrome, fibromyalgia, osteoarthritis, tensional cephalea, endometriosis, or chronic lumbar pain show hyperexcitability after stimulation of healthy tissues. Based in this principle, the system proposed in [229] used an electrical stimulation device that generated a train of five pulses of constant current level, with 1-ms length and repeated at a rate of 200 Hz. As a pain threshold, the lowest current amplitude evoking pain sensing was established, setting the stimulation 1.5 times the value of the threshold. The activity of the anterior tibial muscle was recorded using sEMG. Results showed a high success rate for the correct evaluation of chronic patients compared to healthy volunteers (80% success).

EMG signals have also been used in some pain treatment interventions. An example is the study developed in [230], which proposed an EMG system to provide pain relief for patients with patellofemoral pain syndrome. In these patients, pain is caused by an imbalance between the vastus medialis obliquous and vastus lateral. The system compared EMG signals from both muscles and generated a contraction in the vastus lateral through electrical stimulation that compensated the imbalance.

Another work [231] proposed a facial mask with EMG sensors for the evaluation of facial expressions as the basis of an automatic tool for the objective evaluation of human pain.

### 5.2. Electroencelography and Chronic Pain

EEG is a measurement of biopotentials associated with the electrical activity of the brain by an array of electrodes attached to the scalp. Since EEG signals are very weak, the application of conductive gels can improve the quality of the signal by reducing the impedance of the electrode-skin contact [46]. EEG has numerous practical advantages, since it is a non-invasive technique, with high temporal resolution, provides relevant clinical information, is low-cost and requires little maintenance [50]. EEG studies have shown the activation of specific regions of the brain as a result of pain stimuli [216,217].

Frequency domain analysis, either by fast Fourier transform (FFT), regression models (RM) or wavelet transform (WT) [46], has been employed to study the experience of pain by dynamic analysis of the power in frequency bands [216,218]. Some studies have highlighted the influence of pain in certain frequency bands of the EEG signal, especially in the alpha [50,219] and the gamma [50,232] bands.

Amplitude changes in the alpha frequency band (8–13 Hz) measured at standardized bilateral temporal scalp electrodes (T7 and T8) are associated with tonic pain [216,233]. Power increases in the gamma band (30–100 Hz) have also been related to pain stimuli [216,218]. Since the gamma band is susceptible to higher frequency noise related to EMG signals of facial muscles and artifacts derived from oculomotor activities, adequate signal filtering must be performed [216,234].

In [46], chronic and postoperative pain was analyzed from EEG signals using machine learning techniques. EEG signals were used in [235] (23 channels, a sampling frequency of 256 Hz and 16-bit resolution) to differentiate between harmful (secondary) and benign (primary) headache conditions. EEG has also been employed for the evaluation of chronic pain in general [236] or as an objective marker of pain during the first stage of labor [216].

### 5.3. Electrocardiagram and Chronic Pain

ECG is a biopotential measurement related to the electrical activity generated by the heart [35]. Usually, ECG signal is measured by two electrodes, one on the upper right and one on the lower left of the body, although a third electrode can be used as reference. ECG signal has a characteristic pattern formed by a P wave, a QRS complex and a T signal. Multiple parameters can be extracted from the characteristics of the previous waves, such as the intervals between QRS complexes (R–R intervals), intervals between P waves (P–P intervals), P–R intervals, ectopic pacemakers, etc. However, given the involvement of HRV in the ANS balance and its direct relationship with pain, these parameters have been largely relegated in favor of HRV in the study of pain. For this reason, the fundamentals and applications of HRV for pain assessment were presented in Section 2.

## 6. Computer Vision and Chronic Pain

Computer vision or artificial vision is a scientific/technical discipline that deals with methods for acquisition, processing and analysis of physical world images to abstract relevant information to be managed from a computer. These methods include aspects related to geometry, statistics or information theory. Data correspond to image or image sequences acquired from a video camera or other similar system, such as a medical imaging device.

### 6.1. Recognition of Facial Expressions Associated with Pain

Given that pain response behavior includes facial expressions, possibly intended to request support or empathy [237], computer vision techniques have been used for facial recognition of pain [238]. For this purpose, facial action coding System (FACS) is often used, which is a taxonomy that describes the movements of the facial muscles, to categorize them in a standard and systematic way according to different expressions of emotions, including pain [239]. The implementation of FACS in computer vision systems is performed by detecting geometric features on the face and movement patterns related to facial expressions [240]. Previously, the images must be managed by a procedure for the standardization of image characteristics [54,239]: image segmentation to recognize the face on the background, centering of facial characteristics, normalization of the arguments in the image data, image scaling to predetermined pixel dimensions, etc. If convenient, the image is cropped [241]. The color dimension is usually reduced to black and white. An affine transformation based on face landmarks (mouth corners, nose tip and eyes corners, mainly) is usually employed for face alignment and rotation. The elimination of redundancies and noise from the image allows highlighting the characteristics and structures for a better operation of the facial expression recognition algorithms [239]. Local operations on image texture can also be performed with operators such as local binary patterns (LBP). Discrete cosine transform (DCT) can also be used in the image description process [241]. Other descriptors are based on Taylor series expansion of the image function, named according to the order of the derivative [240,242]. The histogram of the image is a descriptor related to the zero-order coefficient of Taylor series. Other descriptors such as histogram of oriented gradients (HOG) are associated with the first derivative (directional gradient). The speeded up robust features (SURF) and scale-invariant feature transform (SIFT) image descriptors correspond to the second-order derivative of the image (Hessian matrix), which are stable with the image scale and intensity. Finally, the classification is carried out using methods such as those described in Section 8.

The features of face shape have been used for the classification of painful tasks [243] or the evaluation of pain intensity in neonates [244]. The system proposed in [245] used computer vision techniques to analyze the face and the upper part of the body and distinguish among 12 affective states, two of which were fear and anxiety.

A study showed that body and head movements are as effective as facial expressions to detect depression [246]. The system proposed in [247] analyzed images of the movements of sitting and standing, and was able to discriminate between subjects with no pain, subjects with chronic pain, and subjects simulating pain. Differences among the three classes were found in terms of knee, hip and trunk displacements with respect to mean feet support point. In [248], the movements of vertebral deformation for a group of patients with low-back surgery were evaluated through image analysis. With this method, a prediction of pain intensity in a 0-10 scale was reported with a precision of 75%.

### 6.2. Functional Magnetic Resonance Imaging (fMRI) and Chronic Pain

Functional magnetic resonance imaging (fMRI) is another computer vision technique that has been used in the analysis of the central nervous system response in relation to the pain perception and modulation processes, both chronic and acute ones [249,250]. Classification task in these systems is normally based on multivariate pattern analyses (MVPA) [47,251], and on the brain response in two recognizable situations [252]: baseline activity and pain state. Baseline activity is used as reference in the classification method. Magnetic resonance imaging (MRI) sequences are used to construct voxel-based models in activity time series [253]. Images are previously preprocessed to eliminate motion artifacts, breathing and cardiac activity [252]. Through a standardized brain atlas, the brain is segmented into regions of interest (ROIs). The average value of the activity of each ROI is evaluated, and a covariance matrix is constructed [251]. Sparse graphical models (gLASSO) can then be used to reduce the dimensions of the space and estimate the characteristics by partial correlations (inverse of the covariances) [249,250,251]. Structural properties such as volume, surface, curvature or thickness can then be estimated [254].

The characteristics of white matter (axonal integrity) or gray matter (integrity of neuronal bodies) are related to the structural characteristics of the brain assessed through fMRI [255]. Using this technique as a basis, alterations in white matter have been related to different chronic pain conditions [256,257]. On the other hand, a lower density of gray matter in the dorsolateral prefrontal cortex, a region responsible for the modulation and recognition of pain, has been correlated with the chronic low back pain [257,258,259]. On the other hand, fibromyalgia has been related to alterations in gray and white matter volume in certain areas of the brain [260]. Another study in older people has shown an association between chronic pain and brain atrophy [44].

### 6.3. Functional Near-Infrared Spectroscopy (fNIRS) and Chronic Pain

Another neuroimaging method is functional near-infrared spectroscopy (fNIRS) [45]. This method estimates the concentration of oxygenated hemoglobin (HbO) and deoxygenated hemoglobin (HbR) in real time using photoelectric methods. Light absorption rate of HbO and HbR is evaluated at two different wavelengths, typically 695 nm and 830 nm [261]. HbO light absorption is higher than HbR absorption at 830 nm. At 695 nm, just the opposite occurs. From the ratio of light received in the heart pulse related components of both wavelengths it is possible to establish the metabolic changes and the hemodynamics of brain activity [45]. The spatial resolution is obtained through a multichannel optical system that is distributed in matrix form over both hemispheres of the head. Vascular dilation increases brain blood flow, causing the activation of cortical regions. In this way, neuronal activity can be related to fluctuations in the hemodynamic response [262]. Different approaches can be subsequently used for the extraction of neurological characteristics, in time, frequency or time-frequency domains (e.g., wavelets) [48].

fNIRS is a non-invasive technique that has shown to be very useful in the evaluation of brain activity in response to pain stimuli. In [263], fNIRS was proposed as a biomarker of pain in conjunction with machine learning algorithms. Allodynia and motor loss of fibromyalgia patients has been related to alterations in the upper parietal gyrus bilateral region detected by fNIRS [264]. The analysis of cortical activity has shown that the primary motor cortex, premotor cortex and somatosensory cortex are related to pain perception, which was corroborated by pain studies conducted with fMRI, showing the usefulness of HbO as a possible qualitative pain biomarker [265].

## 7. Other Pain Sensors and Data Fusion

This section compiles other sensing technologies and methods applied to the evaluation of pain, different from those already reviewed, and analyzes the benefits of data fusion.

Respiratory rate sensors have been used for the recognition of emotional states [43]. Negative emotions generally induce an irregular respiratory pattern [78]. Respiratory rate is generally reduced with relaxation, however, exposure to tension situations can lead to the sudden stop of respiration. A fast and deep breathing can denote emotions like rage or fear, but sometimes happiness. A fast surface respiration can indicate a tense situation caused by panic, fear or concentration. Slow and deep respiration indicates a state of relaxation, whereas a slow and surface respiration is associated with abstinence states, depression, and also happiness (under calm situations). This way, respiratory sensors might be used for the evaluation of pain, insofar as breathing influences the emotional state [43,78].

According to [108], the irritable intestine syndrome is associated with a higher blood pressure compared to healthy subjects. A higher duration of the syndrome (chronicity) is related with an increase of systolic arterial pressure. Pain chronicity in these patients has also been associated in [28] with a blood pressure drop, a reduction of tachycardia associated to a lower seriousness of the disease (mechanism of “resistance to pain”) and a reduction of bradycardia associated with a higher seriousness of the disease (mechanism of “pain decompensation”).

Skin temperature varies during stress episodes, which can occur in presence of pain situations. As muscles are in a low-tension state, blood vessels contract and cause temperature reduction. However, like conductivity, skin temperature is also influenced by external factors, and it responds slowly to emotional state changes [43].

Blood pulse volume is a measure that allows the determination of the quantity of blood circulating in blood vessels. Photopletismography is based on the emission of light on a body area irrigated with blood vessels, usually a finger [43,266]. A photodetector acquires the light reflected, which depends on the blood volume. Therefore, this technique can be used to measure vasoconstriction, which may be related to pain states.

The mechanical impedance (stiffness) of arterial walls is calculated on a beat-to-beat basis through blood pressure and photopletismography measurements. Results reported in [30] suggest that the changes of stiffness levels can be used to quantify pain level. On the other hand, bioimpedance spectrometry (BIS) is an objective non-invasive tool that has been applied to the diagnostic of knee osteoarthritis [267].

In [268], a biosensor was proposed for the evaluation of pain intensity based on the detection of inflammation markers such as Prostaglandine E2, Cyclooxygenase (COX−1 and COX−2), Leucotrien ($LTA_{4}$ and $LTB_{4}$) and Troponin-C. These measurements are routinely performed in blood analysis laboratories, using specialized equipment based on enzyme-linked immunosorbent assay (ELISA). This immunoassay technique detects an immobilized antigen through an antibody tied to an enzyme that generates a detectable product or a change of color in the sample. The procedure involves getting a blood sample of the patient, the preparation of reactives, antibody solution, substrate solution, and incubation with light protection, light exposure and correction of wavelength. The process takes approximately one week to obtain the final report. The proposed biosensor requires a blood sample and estimation can be done in a shorter time, although the necessary equipment is complex and costly, as it requires microelectronic technologies, the immobilization of antygens on a substrate, the use of laser beam and a photodetector.

The smart chair described in [269] uses capacitive sensing to estimate respiratory rate, recognize the performed activity and detect inappropriate postures that can unleash chronic back pain.

The analysis of gait has also been related to pain [270]. A study during pregnancy and postpartum with pressure sensors located in instrumented foot insoles correlated the prevalence of low back pain with alterations in the gait: increased pressure in the hindfoot and excessive pronation [270].

Other approaches employ data fusion from different sensors. These methods are beneficial because they allow a complementary and redundant use of information that can provide a more accurate and reliable result [271,272,273]. Data fusion can reduce uncertainty in the evaluation stage and reduce the risk of loss of information, noise or errors in measurements of a particular sensor. On the other hand, complementary information from multiple sensors allows access to environmental characteristics that would not be possible to perceive in a single sensor approach [271].

Data fusion at the processing level can be considered at two levels [54]: early fusion, by combining different categories into a single data stream; or late fusion, by integrating outputs of individual classifiers as inputs to the next level of classification.

Multiple approaches to data fusion have been proposed in the literature. In [274], audio, video, temperature, ECG, EMG, and skin conductance signals were used to better pain classification. Sickle cell disease patients experiencing chronic pain were evaluated with heart rate, accelerometry, gyroscopy, temperature and skin conductance sensors [275]. In [276] and in [49], EEG and fMRI data fusion provided an improvement in accuracy. Patients with sickle cell disease were evaluated in [277] using systolic and diastolic blood pressure, peripheral capillary oxygen saturation, respiratory rate, heart rate and temperature sensors. In [278], chronic pain using heart rate and blood pressure sensors was evaluated. Signals of skin conductance levels, EMG and ECG were fused in [51] to analyze the response to thermal pain induced in healthy patients.

The system described in [279] proposes a multisensory approach (accelerometry, photoplethysmography (PPG), body temperature and electrodermal activity) to detect a migraine event in a previous state in order to notify patients to take their medications with enough time. In [280], another multisensory approach was used, based on acceleration and electromyogram sensors, to assess low back pain.

Pain intensity induced thermally was recognized in [27] through the joint use of video and audio signals (respiration sounds and sporadic moans acquired by a headphone located in the nasolabial region).

Finally, and although it is not directly related to the detection of pain, but to its avoidance, it is worth mentioning a system for the recognition of the distribution and imbalances of the weight of backpacks, providing feedback to the user to avoid problems related to back pain [281].

## 8. Processing Algorithms and Computational Models for Pain Assessment

As the evaluation of chronic pain is a complex task that often poses a challenge due to the amount of data to be managed, especially in the case of facial expression images, MRI, and multisensory approaches combining different approaches described so far, in recent years numerous machine learning algorithms have been proposed [253,255,282]. In a generic way, pain assessment is usually performed through procedures in which the following main stages can be distinguished:

### 8.1. Preprocessing

A preprocessing is applied to physiological signals to remove unwanted artifacts [51]. This preprocessing can be based on band-pass filters to remove motion artifacts and drifts from the continuous level of the signal, associated with the low-frequency components [49,50], or noise, normally included in the high-frequency components [35,50]. The electromagnetic interference component associated with the power grid can be removed by a notch filter at 50/60 Hz.

### 8.2. Feature Extraction

In the feature extraction stage, an abstraction of the relevant information from larger data elements of the physiological signal is performed [51,251,275]. The objective of this stage is to improve the density of information. Data extraction may be related to characteristics of the signal in the temporal domain, but also in the frequency domain [49]. Some features may be amplitude, frequency, stationarity, linearity, variability, similarity, entropy, etc. [35,51]. The features can in turn be standardized to keep all parameters in a comparable range of values [35].

### 8.3. Feature Selection

To decrease the computational cost, the subset of features that provide meaningful information related to the pain classification problem is selected [51,275]. In this way, noisy or redundant features can be removed for the subsequent analysis [251]. The objective of this stage is to improve the quality of the information. Univariate feature selection (UFS) implies the selection of features through a machine learning algorithm that iteratively improves a quality criterion (correlation coefficient, analysis of variance (ANOVA) F-value, mean square error, etc.) to yield a desired threshold [35,51,251]. In contrast, in sequential forward selection (SFS), the final classifier is used in the selection of the best features [251,275]. Principal component analysis (PCA) can also be used to reduce the number of features [216,283].

### 8.4. Classification

After extracting features, the pain classification algorithm takes place, composed of a first stage for pain assessment model training and a second stage for the test [35,251]. A common approach is to use a cross-validation, in which one part of the data is used to train the model, and another part is dedicated to the test, both independent from each other to model a realistic error [251,275]. Data augmentation is also often required to increase the number of entries used in the training process [239]. Some of the models that have been used for pain assessment are the following (at the end of each model some successfully studies related to the evaluation of chronic pain are shown):

- K-nearest neighbors (kNN): In this method, the output is the class resulting in the classification, which is obtained by a majority of votes of its closest *k* neighbors: [277,284].
- Support vector machine (SVM): In SVM model the data are represented as points in a hyper-space of high dimension. The optimization process tries to find the hyper-plane that defines the separation surface between categories, maximizing the distance from all points: [35,46,51,54,258,277,283,284,285,286].
- Multinomial logistic regression (MLR): MLR is a classification method that generalizes logistic regression (a binary regression model that uses the logistic function to model the binary dependence) with a multiclass problem. The MLR model will finally be the probability of assignment to a certain class, choosing the one that provides the greatest probability: [258,277].
- Bayesian networks (BN): BN is a graphic model that specifies a probability distribution in a set of random variables. It consists basically of two components: a probability distribution of the variables and a directed graph that shows the dependencies between variables: [258,284].
- Restricted Boltzmann machine (RBM): It is a graphic model with symmetric connections between observable and hidden variables to model a probability distribution. The model is called restricted because it has no connections between elements of the same layer, which favors the learning process: [278].
- Relevance vector machine (RVM) and Gaussian process (GP) models: RVM and GP are Bayesian extensions of the SVM algorithm, that allow assessing the pain scale based on the probability of belonging to a class: [244,253].
- Decision tree (DT): DT is a graph-shaped model that represents the decision points as ramifications and the desired prediction as end-nodes or leaves. It is therefore a rule-based model that induces solutions from the decision rules: [258,284,287,288,289].
- Random forest (RF): An RF is a set of DTs, where each DT has a subset of leaf nodes. No tree has all training data. This causes each tree to be trained with different data samples for the same problem. In this way, by combining their results some errors are compensated with others and a more generalized prediction is obtained: [50,290,291,292].

Models based on artificial neural networks (ANNs) have also been used in pain assessment because of their ability to learn, generalize problems and abstract essential characteristics from inputs with apparent irrelevant information [293]:

- Multilayer perceptron (MLP): The multilayer perceptron is an ANN formed by multiple layers of a basic unit or artificial neuron known as perceptron, with a forward directed flow of information from the input layer to the output layer, passing through the hidden layer/layers, and with a learning algorithm through backward propagation: [258,284,293,294]
- Deep learning (DL): DL is based on a division in multiple levels that correspond to different levels of abstraction for data expressed in matrix form: [295].
- Convolutional neural networks (CNNs): This ANN is an extension of MLP applied to two-dimensional matrices through a convolution operation [295], more suitable for computer vision tasks. In an MLP, the output of each neuron depends on all inputs. This structure does not allow the network to model patterns such as those that can be found in an image. CNN neurons are connected to a small number of inputs belonging to a small continuous region [293]. Convolutional kernels are used to extract multiple local characteristics and adjust to different patterns. This characteristic considerably reduces the number of parameters and allows a greater specificity in the recognition of characteristics: [52,239,293,295,296,297,298,299,300].
- Deep belief networks (DBNs): It is a type of DL with connections between layers but not between units within the same layer: [239].
- Recurrent neural networks (RNNs): This type of ANN represents a directed graph capable of abstracting temporal sequences of data, providing the ability to model dynamic behaviors: [239,293].
- Mixed networks (MNs): They are hybrid networks formed by the interconnection of two or more different ANNs, such as CNN and RNN: [54,239].

Table 1 shows a summary of some of the results obtained with machine learning techniques in pain assessment studies (pain classification accuracy defined according to [284]).

## 9. Digital Resources for the Evaluation of Pain

Finally, this section compiles diverse digital resources (database, mobile apps, etc.) developed in the context of chronic pain detection [301,302].

The system proposed in [37,303] recognizes physical pain situations under rehabilitation using high-resolution video images for the capture of facial expressions, a system for the 3D acquisition of full-body movements (Animazoo IGS-190) and four EMG sensors (BTS FREEEMG 300) placed symmetrically on the upper trapezius fibers and lumbar para-spinal muscles. The acquired data are available for the scientific community through a web link [304]. This database has been used for the chronic lumbar pain recognition through the detection of behaviours related to pain, such us guiding actions or sudden movements [7]. It has also been used for the automatic detection of protecting behaviours [305] or the classification of pain levels (“none”, “high”, “low”) during rehabilitation process [306]. The same records were used in [11] to discriminate low-intensity from high-intensity pain in full-trunk flexion movements (94% success), and sit/stand activities (80% success).

Pain situations were detected in [307] using UNBC McMaster database [308], with a positive-trues rate of 82.4%. This database contains video sequences of spontaneous facial expressions for patients with shoulder pain. The same database has been used in other studies, like [20], which reported a system that recognizes four pain intensities, or [29], which calculated a continuous variable related to pain intensity. The system presented in [309] established the probability of future pain considering current pain state.

Another database for pain research is BioVid Heat Pain [42,43]. This database includes video images, EMG, ECG, and skin conductance records from 90 volunteers under normal situation and heat-induced pain. This database was used in [23] to develop a detection system that obtained a success of 80%.

SenseEmotion is a database generated by the Department of Psychosomatic and Psychotherapy Medicine of Ulm University [19]. It includes signals acquired with diverse sensors (skin conductance, ECG, EMG, respiratory rate, video recording of facial expressions, and audio recordings), which were obtained from volunteers undergoing a set of random thermally-induced pain experiments.

X-ITE Pain Database will provide sensory information from multiple sensors (video, audio, skin temperature, ECG, skin conductance, EMG, among others) obtained from subjects undergoing stimuli of different intensity (low, medium, and high), duration (5 s/1 min) and mode (electric/heat pain) [274].

**Figure 8:**
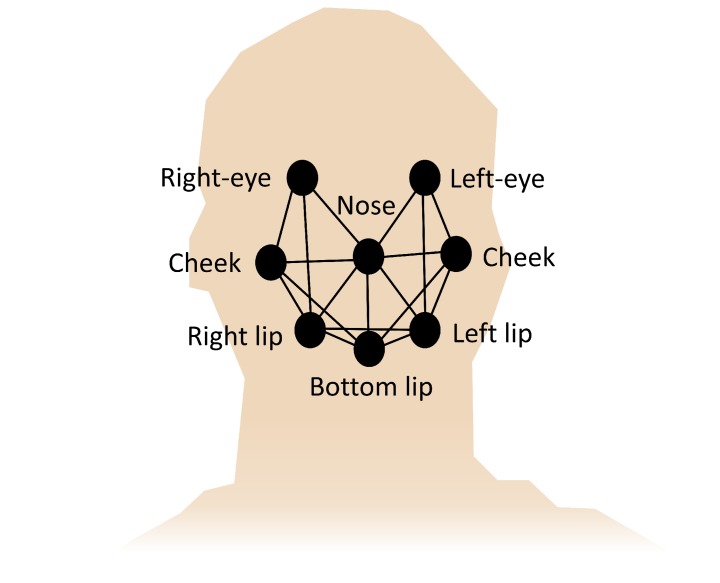
Example of landmarks extracted by Google’s Face API according to [314].

Different applications have been proposed to be executed in smartphones. Pain Care [310] is a mobile app that assists patients with chronic pain in the management of their symptoms, drugs and communication with medics. Another example is the app described in [311], which performs an evaluation of pain using facial images acquired with a smartphone. Images are decomposed in vector subspaces with sub-images as characteristic vectors defining the image. The mobile app [3] allows the classification of pain level (“normal”, “moderate”, “severe”) using information from different sensors (heart rate, skin conductance, respiratory rate and skin temperature). Another app has been used to assess the level of wrist movement, which was inversely related to the degree of wrist pain self-reported by the patient [312]. In [313], an app is described that uses machine learning on physical activity data and pain self-report documents to find routine behaviors and automatically generate physical activity recommendations. The app proposed in [314] is aimed at the automatic detection of pain by recognizing spontaneous facial expressions using Google’s Face API. Figure 8 shows an example of facial landmarks extracted by Google’s Face API that are employed in the app.

In [315], the authors propose a virtual reality (VR) environment for the motivation of patients in therapies for chronic pain relief. Another example of how biomedical engineering and virtual reality (VR) can help chronic pain relief is shown in [316].

## 10. Future Trends and Conclusions

Pain assessment is critical in a wide range of disorders [20]. The prevalence of chronic pain is global [313]. This condition is closely related to the state of psychological well-being, and is often in close union with anxiety problems, depressive disorders and negative affectivity [313]. Subjective and self-reported account of patients, caregivers or medical staff are currently the common method to measure pain [21]. However, self-assessed reports have limitations due to their subjective nature that can lead to inadequate and insufficient therapy [20,27].

Although the objective measurement of pain using biomedical sensors has been raised as a promising approach to address this problem, the precise measurement of pain remains a challenge for researchers and healthcare professionals [28]. Objective pain assessment has been investigated from multiple approaches. HRV has been used for the evaluation of chronic pain of different etiology (headache, neck, shoulder, lower back, fibromyalgia, hip, gastrointestinal, burning mouth syndrome, etc.) [28,77,91,92,93,94,95,96,97,105,109]. Different studies have shown that people with chronic pain have decreased HRV compared to people without pain, especially in the high frequency component [77,95].

On the other hand, it is common that patients who feel chronic musculoskeletal pain are less physically active [123]. The anxiety produced by the feeling of pain causes patients to behave in a self-protective manner, avoiding the type of movement that causes the pain [11]. However, and despite all these conditions, the measurement of physical activity remains a challenge in the treatment, rehabilitation, and health promotion of patients with chronic non-oncological pain [131]. Physical activity monitoring devices have the potential to help clinicians in multiple novel pain applications, such as surgeon support tools to provide personalized post-operative care [317].

Additionally, a greater research effort is required to understand the mechanisms related to movement deficits in patients with pain, including monitoring processes through sensor technologies in routine clinical protocols [129]. More studies are needed to analyze the characteristics of movements during gait or posture and their correlation with pain. These studies can be used to design exercise programs for preventive treatment and pain rehabilitation [270].

Frequent physical activity can attenuate pain level in the case of chronic musculoskeletal pain, however, patients with pain tend to limit physical activity as a result of the psychological association established between pain and movement [313,318]. Self-reported pain and movement disability are directly related to the natural tendency to avoid pain [125]. Despite the research progress, more studies are needed that relate the kinematics of body movements to pain avoidance mechanisms [125]. To achieve this, an external feedback mechanism is essential to allow patients to learn and correct their movement habits.

On the other hand, the monitoring of physical activity may be affected by circumstances of personal mood, weather conditions, and other psycho-social factors [25]. Future studies should incorporate these aspects to provide more reliable measures of physical activity, such as variability or regularity in the development of activities, and symmetry of gait [25]. The effect of physical activity on pain is very broad, and new studies are necessary to incorporate an analysis of the frequency, intensity, time, and type of physical activity [174]. These studies should be evaluated by classifying patients into subgroups, according to their respective activity limitations.

Skin conductance has also been applied to the evaluation of pain, since there can be a rapid increase in its value as a result of an increase in the secretion of sweat glands, innervated by the sympathetic branch of the ANS.

Other systems used in pain assessment are based on EMG signals, since a high level of muscle tension is an indicator of pain [35,43]. Facial EMG is an alternative technique to assess pain perception through facial muscle activity [319]. However, deeper studies are necessary to prove the correlation with pain.

Image processing and computer vision techniques have also been used in the recognition of pain [243] or the evaluation of pain intensity [244]. Facial movements associated with pain feeling can be identified with computer vision techniques [319]. This procedure has some limitations, such as the movement artifacts of subjects that must be addressed before the translation to clinical practice [319]. Other problems are the complexity of image-based systems and the cost of the devices. Important research challenges are posed to mitigate the aforementioned limitations. The processing of audio signals can also be useful by means of the detection of noise and groans related to pain [27].

Other parameters have also been used in the evaluation of pain, such as the monitoring of respiratory rate [43], since negative emotions generally induce an irregular respiratory pattern [78]. Blood pressure has also been a study parameter, which may be higher in pain situations [108]. The body temperature has also been considered, since in situations of pain the muscles are under tension, the blood vessels contract and, therefore, the temperature decreases [43]. Photoplethysmography can also be used to measure vasoconstriction, which may be related to pain states [43,266].

However, there is still no universal method that allows the objective evaluation of pain [22], its intensity or severity, although it may be critical in multiple conditions [28].

One of the major limitations hindering the objective evaluation of pain is the lack of studies with clinically valid data [320]. It is necessary to incorporate biomarkers in the evaluation studies that allow to establish correspondences with a reference standard, such as yclooxygenase-2 or the inducible nitric oxide synthase [320]. Further research is necessary through randomized clinical trials on different populations and considering different therapeutic modalities to confirm the results of previous studies and provide more evidence on the possibility of objectively assessing chronic pain with sufficient precision to be useful toward a more personalized, proactive and preventive medicine [321].

The measurement of pain in non-communicative patients is a research challenge [231,319], which extends from infants and children [322] to adults in the post-operative period, in intensive care units or with cognitive impairment. An application of great interest is the closed-loop peri-operative monitoring with the purpose of automatically controlling the administration of anesthesia [319].

People who work in a closed environment and sit in a chair sometimes maintain inappropriate postures that can lead to damage and affections in the spine [323]. Some proposals based on accelerometers and gyroscopes have been made to detect incorrect postures and provide tactile feedback through vibrations to notify the user of the need to correct the posture. Low back pain has a great impact on work productivity worldwide, despite years of research in this area [280]. Understanding the mechanisms involved in the injuries that cause pain can be useful to improve prevention and treatment in occupational risk protocols. New solutions based on feedback mechanisms to users would be highly recommended, which can alert the user when they remain in a position for a long time or when they make inappropriate movements [280]. The feeling of security that could be assumed by the subject would improve their quality of life and the well-being of the worker and would have a positive health impact since harmful positions would be avoided.

Rehabilitation is essential in the control of multiple musculoskeletal conditions [132] related to pain. Rehabilitation treatment is largely based on the performance of a series of exercises in the home environment, but on many occasions patients do not adequately comply with the prescribed exercise and the degree of compliance with the rehabilitation program is not known. There is a need for technological tools that allow the supervision of rehabilitation therapies in patients’ homes, positively influencing the motivation and control of patient adherence [132]. Physical rehabilitation must be adapted by physiotherapists according to the movement abilities of patients [124]. In this sense, technology can provide fundamental support in physical rehabilitation, to the extent that it would allow the evaluation of patient movement patterns to provide such personalized follow-up [124].

In the rehabilitation process, the ability to provide visual and acoustic feedback to the patient to promote their motivation in compliance with the prescribed rehabilitation program should be investigated [129]. The aspects related to the retro-feedback of patients remain unexplored. It would be of great interest to reinforce research into automated technological solutions that enable positive beliefs, stimulation and self-efficacy of physical activity, since the overload of medical staff prevents the patient from being treated in a holistic and continuous way [313]. Future studies should also analyze the relationships between the improvement produced in pain and the mechanisms of intervention and feedback to patients [280]. In these studies, a multi-causal approach should be considered in order to distinguish between the benefits directly caused by the reduction of pain from other additional psychosocial factors that can cause similar or placebo effects [280].

The non-intrusiveness of the measurements carried out by the sensor devices during the subject’s daily life is another challenge to be approached in future research [169]. The same concerns apply to the development of pain assessment technologies through the miniaturization of the devices to achieve true continuous monitoring 24 hours a day [266].

Although many studies have evaluated pain by observing individual physiological parameters, future studies should employ a multisensory point of view to allow a deeper understanding of the causes and effects of pain [280]. However, a balance must be established between the complexity and cost of the systems with respect to the benefits provided in precision. Future studies should explore the cost-effectiveness of the systems and the possibilities of inclusion in the clinical guidelines for patient healthcare [280]. These research studies should focus on randomized clinical trials over larger populations, also incorporating an economic evaluation [280].

The incorporation of the new technologies in pain assessment systems is another area of great development potential, either supported by smartphones or smart watches, to the extent that many of these systems already incorporate means for the evaluation of physiological signals such as heart rate or physical activity in real time [301]. Smartphones can be used to collect the results of pain self-assessment reports more efficiently [312]. In turn, the sensors incorporated in smartphones can be used to gather clinical information of relevance, which may be of particular interest in the evaluation of pain [312]. This approach can be expanded with the incorporation of social networks [312]. Internet-of-Things (IoT) is a recent area of study of great popularity and objective pain assessment can be benefited by this ubiquitous monitoring approach [302].

Artificial neural networks (ANNs) are currently considered a powerful method for data analysis and pattern recognition [324]. In particular, the application of so-called deep learning neural networks have grown with great intensity in recent years [325]. The design of the architecture of these networks remains an open area of study and their application to the objective evaluation of pain is clearly justified when physiological signals of different etiology are evaluated [324]. The objective measurement of pain through biomedical sensors is not ready for clinical use, hence there are enormous research and innovation opportunities in the field of the application of sensor technologies combined with artificial intelligence and machine learning techniques [319].

A greater research and development effort in health technologies is necessary, both in private and public assistance models, including rural areas and taking into account the vision and needs of the pain-affected patients [317]. There is a need for new systems and applications for chronic pain self-control, incorporating the needs and context of the user, which would allow improving accessibility to treatment, and reducing economic costs and waiting times [314]. However, current pain management developments have not been designed with integrated features that effectively address the multidimensional nature of pain.

Acute and chronic pain are two different clinical conditions of pain [326], however, many studies do not discriminate between acute and chronic pain [327], partly because among the challenges to both researchers and clinicians is the understanding and distinction between acute and chronic pain [328]. Although many studies address acute nociceptive pain, sometimes performed by phasic pain stimuli (short duration), the number of studies based on tonic pain stimuli is growing as an approach to chronic pain [41,42,44,51,52]. This validation approach represents an objective way for assessing chronic pain, leaving aside all the subjectivity that may be related to the perception of pain by the chronic patient. Chronic pain is in general more challenging than acute pain, but also has a larger impact on society, so that research into chronic pain assessment methods of different etiology should be enhanced.

More studies are needed to analyze the effects of treatments and interventions on pain, as well as their functional manifestations, which would allow to optimize physical and clinical interventions on patients suffering from pain [24]. For this purpose, the integration of the information from these monitoring systems with the health systems in a standardized manner is necessary and remains a challenge for researchers and developers [301].

In conclusion, objective sensor-based pain monitoring technologies have the potential to help improving the patient’s quality of life [312], to understand better the pain biopsychosocial processes and may be of interest in the study of other related conditions [172].

## Figures and Tables

**Table 1:** Processing algorithms examples for pain assessment.

Study	Pain Etiology	Machine Learning Model	Sensor Data	Pain Classification Accuracy
[239]	Shoulder pain	CNN, RNN	Facial expressions (computer vision)	75%
[255]	Chronic low back pain, osteoarthritis and fibromyalgia	SVM	fMRI	75%
[241]	Infant pain	SVM	Facial expressions (computer vision)	83.8%
[45]	Induced pain in healthy people	k-NN	fNIRS	92.1%
		SVN	fNIRS	91.2%
[290]	Musculoskeletal chronic pain	RF	Electronic health records	94%
[244]	Infant pain	RVM	Facial expressions (computer vision)	91%
[252]	Chronic low back pain	SVM	fMRI	92.5%
[258]	Fibromyalgia	DT	fMRI	76%
[48]	Induced pain in healthy people	K-NN	fNIRS	88.3%
[216]	Shoulder pain	SVM	EEG	84%
[298]	Multiple pain etiology	CNN	Facial expressions (computer vision)	93.3%
[283]	Pain after surgery	SVM	Facial expressions (computer vision)	87%
[286]	Pain after surgery	SVM	Skin conductance	77.7%
[299]	Shoulder pain	CNN	Facial expressions (computer vision)	98.5%
[300]	Infant pain	CNN	Facial expressions (computer vision)	88.3%
[49]	Induced pain in healthy people	SVM	EEG	78.2%
[50]	Induced pain in healthy people	RF	EEG	89.5%
[277]	Sickle cell disease pain	k-NN, SVM	Multisensor	68%
[278]	Multiple chronic pain	RBM	Heart rate, blood pressure	72%
[52]	Induced pain in healthy people	CNN	EEG	97.4%
[51]	Induced pain in healthy people	SVM	EMG, skin conductance, ECG	79.4%
[54]	Neck and shoulder pain	SVM	EMG	77%

## Abbreviations

The following abbreviations are used in this manuscript:

ANN	Artificial Neural Networks
ANOVA	Analysis of Variance
ANS	Autonomic Nervous System
API	Application Programming Interface
BIS	Bioimpedance Spectrometry
BN	Bayesian Networks
BPAQ	Baecke Physical Activity Questionnaire
BSN	Body Sensor Networks
CNN	Convolutional Neural Network
DBN	Deep Belief Networks
DCT	Discrete Cosine Transform
DL	Deep Learning
DT	Decision Tree
ECG	Electrocardiagram
EEG	Electroencephalogram
ELISA	Enzyme-Linked Immunosorbent Assay
EMG	Electromyogram
EMI	Electromagnetic Interference
FACS	Facial Action Coding System
FFT	Fast Fourier Transform
fMRI	Functional Magnetic Resonance Imaging
fNIRS	Functional Near-Infrared Spectroscopy
gLASSO	Sparse Graphical Model
GP	Gaussian Process
HbO	Oxygenated Hemoglobin
HbR	Deoxygenated Hemoglobin
HF	High-Frequency
HOG	Histogram of Oriented Gradients
HRV	Heart Rate Variability
Iot	Internet of Things
k-NN	k-Nearest Neighbors
LBP	Local Binary Patterns
LF	Low-Frequency
MLP	Multilayer Perceptron
MLR	Multinomial Logistic Regression
MN	Mixed Networks
MORE	Mindfulness-Oriented Recovery Enhancement
MPQ	Mcgill Pain Questionnaire
MRI	Magnetic Resonance Imaging
MVPA	Multivariate Pattern Analyses
PCA	Principal Component Analysis
PPI	Present Pain Intensity
PRI	Pain Rating Index
PSD	Power Espectral Density
PPG	Photoplethysmograph
RBM	Restricted Boltzmann Machine
RF	Random Forest
RM	Regression Models
RNN	Recurrent Neural Networks
ROI	Regions of Interest
RVM	Relevance Vector Machine
Semg	Surface Electromyogram
SFS	Sequential Forward Selection
SIFT	Scale-Invariant Feature Transform
SURF	Speeded Up Robust Features
SVM	Support Vector Machine
UFS	Univariate Feature Selection
VAS	Visual Analog Scale
VLF	Very-Low-Frequency
VR	Virtual Reality
WT	Wavelet Transform
